# Development and internal validation of a 5-year major cardiovascular and cerebrovascular events prediction model using a simplified carotid plaque score in a hospital-based primary-prevention cohort

**DOI:** 10.3389/fcvm.2026.1966647

**Published:** 2026-10-28

**Authors:** Haitao Hu, Daoyan Li, Qian Zhang

**Affiliations:** Department of Ultrasound, Cardiovascular and Cerebrovascular Disease Hospital, General Hospital of Ningxia Medical University, Yinchuan, China

**Keywords:** cardiovascular risk prediction, carotid plaque, carotid ultrasonography, major adverse cardiovascular events, subclinical atherosclerosis

## Abstract

**Background:**

Carotid plaque burden is an established cardiovascular risk marker, but the incremental predictive value of a simplified six-segment plaque score within a multivariable model remains less well characterized in hospital-based Chinese primary-prevention populations. We aimed to develop and internally validate a 5-year model for major adverse cardiovascular and cerebrovascular events (MACE) incorporating this score.

**Methods:**

This single-center retrospective cohort included adults aged ≥40 years without prior clinical cardiovascular or cerebrovascular disease who underwent bilateral carotid ultrasonography between 2015 and 2020. Plaque presence in six carotid segments generated a 0–6 score. The primary outcome was 5-year hard MACE, defined as cardiovascular death, nonfatal myocardial infarction, or nonfatal ischemic stroke. Three Cox models were compared: clinical predictors alone, clinical predictors plus binary plaque presence, and clinical predictors plus plaque score. Performance was assessed by discrimination, calibration, Brier score, decision curve analysis, and 1,000 bootstrap resamples.

**Results:**

Among 2,413 patients, 1,560 (64.6%) had carotid plaque and 108 experienced hard MACE during a median follow-up of 4.92 years. Five-year MACE incidence increased from 1.5% for plaque scores 0–1 to 6.1% for scores 2–4 and 15.4% for scores 5–6 (*P* < 0.001). Each 1-point score increase was associated with higher MACE risk (HR, 1.32; 95% CI, 1.21–1.44). The apparent C-index increased from 0.753 for the clinical model to 0.804 with the plaque score; the corresponding 5-year AUC and Brier score were 0.807 and 0.0381. After bootstrap correction, the C-index was 0.792 and calibration slope 0.93. Model 3 showed greater net benefit across most 2%–10% thresholds, with broadly similar findings in sensitivity analyses.

**Conclusions:**

In this hospital-based primary-prevention cohort, the simplified six-segment carotid plaque score provided incremental predictive information beyond conventional clinical factors and binary plaque presence. External validation, particularly in lower-risk community and primary-care populations, is required before broader clinical application.

## 1 Introduction

Cardiovascular disease (CVD) remains the leading cause of mortality worldwide and continues to impose a substantial burden despite advances in prevention and treatment. In 2022, CVD accounted for an estimated 19.8 million deaths globally, with ischemic heart disease and stroke contributing most of this burden (1). Atherosclerosis develops over decades before the first clinical event, creating an opportunity for earlier risk identification in apparently asymptomatic adults. Carotid atherosclerosis is a common manifestation of this subclinical disease. A global systematic review estimated that, among adults aged 30–79 years, carotid plaque affected 21.1% of the population in 2020, corresponding to approximately 816 million individuals (2). In China, a nationwide analysis of more than 10.7 million adults reported a carotid plaque prevalence of 21.0%, with substantially higher prevalence among older adults and individuals with hypertension, diabetes, dyslipidemia, or other cardiovascular risk factors (3).

Primary prevention currently relies largely on multivariable risk equations combining demographic and conventional clinical factors. SCORE2, for example, estimates 10-year risk of fatal and nonfatal CVD using age, sex, smoking status, systolic blood pressure, and lipid measures and was derived from more than 670,000 participants across multiple European cohorts (4). Although such models provide an essential framework for prevention, individuals with similar conventional risk-factor profiles may have markedly different burdens of established subclinical atherosclerosis. Imaging-based assessment can therefore provide complementary information by measuring the vascular consequences of cumulative risk exposure. A systematic review of asymptomatic low-to-intermediate-risk populations found that carotid plaque detection and quantification can improve cardiovascular risk characterization beyond conventional risk assessment, although methods of plaque assessment and study designs were heterogeneous (5). In the Multi-Ethnic Study of Atherosclerosis, carotid plaque presence and plaque score were independently associated with long-term coronary heart disease risk even among individuals with a coronary artery calcium score of zero, further supporting the prognostic relevance of carotid plaque burden (6).

However, translating carotid imaging into routine risk prediction remains challenging. Prior studies have used binary plaque presence, maximal plaque thickness, total plaque area, plaque counts, or more complex imaging phenotypes, which differ in acquisition burden and reproducibility and may limit implementation across routine ultrasound laboratories (5, 6). A simple score based on the number of plaque-positive carotid segments may offer a pragmatic alternative. Recent analyses from the Multi-Ethnic Study of Atherosclerosis and Atherosclerosis Risk in Communities cohorts used a harmonized 0–6 carotid plaque score based on bilateral common carotid, bifurcation, and internal carotid sites and demonstrated its relevance to cardiovascular risk stratification and primary-prevention decision making (7). These and other studies support the prognostic relevance of carotid plaque extent. A remaining practical question is how such a simplified segment-based measure performs when incorporated into a multivariable prediction model using routinely available clinical variables, including its incremental discrimination, calibration, prediction error, and potential clinical utility in distinct real-world populations.

Accordingly, this study aimed to develop and internally validate a 5-year risk prediction model for hard major adverse cardiovascular and cerebrovascular events (MACE) incorporating a simplified 0–6 carotid plaque score in hospital-based adults without previous clinical cardiovascular or cerebrovascular disease who had already undergone carotid ultrasonography. The intended use of the model was risk stratification after carotid ultrasound obtained during health evaluation, cardiovascular risk assessment, or stable chronic disease management; it was not developed to determine eligibility for carotid ultrasound or for population-wide cardiovascular screening. The primary methodological focus was to quantify the incremental predictive information provided by plaque extent beyond conventional clinical predictors and binary plaque presence, with assessment of discrimination, calibration, overall prediction error, clinical net benefit, internal validity, and robustness across sensitivity analyses.

## 2 Methods

### 2.1 Study design

This single-center retrospective cohort study was conducted to develop and internally validate a prediction model for the 5-year risk of MACE. The source population comprised hospital-based adults without established clinical atherosclerotic CVD who underwent bilateral carotid ultrasound at General Hospital of Ningxia Medical University for routine health evaluation, cardiovascular risk assessment, or stable chronic disease management between January 1, 2015, and December 31, 2020, with outcome ascertainment through December 31, 2025. For patients with multiple eligible examinations, only the first qualifying ultrasound was included, with its date serving as the index date. Baseline clinical, laboratory, and sonographic data obtained on or before the index date were retrieved from the hospital information system, electronic medical record (EMR), laboratory information system, picture archiving and communication system (PACS), and regional mortality registry. The study protocol was approved by the Medical Ethics Committee of the General Hospital of Ningxia Medical University (Approval No. KYLL-2026-1414), which waived the requirement for informed consent. The study was conducted in accordance with the Declaration of Helsinki.

### 2.2 Study population

The study cohort was identified by querying all bilateral carotid ultrasound examinations recorded in the institutional EMR and PACS between January 1, 2015, and December 31, 2020. Ultrasound records were linked to demographic data, diagnoses coded according to the International Classification of Diseases, 10th Revision (ICD-10), procedure histories, and available longitudinal clinical records. Candidate eligibility was initially screened electronically and subsequently confirmed by manual review of the index encounter and medical history. Uncertain clinical eligibility was adjudicated by a senior physician, and equivocal image adequacy or segment-level plaque status was reviewed by an experienced vascular ultrasound physician.

Patients were eligible if they were aged ≥40 years at the index examination and underwent bilateral carotid ultrasound during routine health evaluation, cardiovascular risk assessment, or stable chronic disease management in the absence of established clinical cardiovascular or cerebrovascular disease. The index examination was required to contain archived images or a structured report sufficient to determine plaque status in six prespecified arterial segments: the bilateral common carotid arteries (CCAs), carotid bulbs, and proximal internal carotid arteries (ICAs).

Patients were excluded if they had a documented history before the index date of myocardial infarction, established coronary artery disease or chronic coronary syndrome, any previous stroke, transient ischemic attack (TIA), symptomatic peripheral artery disease, or prior coronary, carotid, or peripheral arterial revascularization (8). Carotid ultrasound examinations performed as part of the diagnostic evaluation of an acute or suspected acute cardiovascular or cerebrovascular event were also excluded. Additional exclusions were insufficient ultrasound information to determine plaque status across all six prespecified segments, inability to reliably establish patient identity, or absence of any post-index clinical record or registry-linked information permitting outcome ascertainment.

### 2.3 Data collection and definitions

Clinical, laboratory, and sonographic data were extracted using a standardized electronic case report form. A random 10% sample of records was independently reviewed by a second researcher for data quality control. The primary indication for carotid ultrasonography was abstracted from the ultrasound order and index-encounter documentation and classified as routine health evaluation, cardiovascular risk assessment, or stable chronic disease management. When more than one indication was documented, the indication recorded as the principal reason for the examination was used. Referral source was classified according to the ordering service as the health management/physical examination center, cardiovascular medicine, cerebrovascular/neurology service, or other internal medicine service. Inpatient or outpatient status was determined from the index encounter associated with the ultrasound examination. Body mass index (BMI) and systolic blood pressure were obtained from measurements recorded on the index date whenever available; otherwise, the most recent measurements within the preceding 30 days were used. Laboratory variables were based on the most recent measurements obtained within 30 days before or on the index date. Estimated glomerular filtration rate (eGFR) was calculated uniformly from serum creatinine, age, and sex using the 2021 Chronic Kidney Disease Epidemiology Collaboration (CKD-EPI) creatinine equation (9).

Segment-level carotid plaque status was abstracted from structured ultrasound reports and verified against archived PACS images when the report was incomplete or ambiguous. Plaque was defined according to the Mannheim consensus as a focal structure encroaching into the arterial lumen by ≥0.5 mm, measuring ≥50% thicker than the surrounding intima-media thickness (IMT), or having a focal thickness >1.5 mm from the media-adventitia interface to the intima-lumen interface (10). The simplified carotid plaque score was calculated across the bilateral CCAs, carotid bulbs, and proximal ICAs. Each segment containing plaque was assigned 1 point, yielding a total score ranging from 0 to 6 (11). The score was intended to quantify the anatomical extent of plaque across prespecified carotid segments and was not a measure of luminal stenosis severity or plaque morphology.

Maximal carotid stenosis severity was abstracted from the index carotid duplex ultrasound report. During the study period, internal carotid artery stenosis was classified according to the Society of Radiologists in Ultrasound consensus criteria (12), integrating gray-scale and color Doppler assessment of plaque and luminal narrowing with spectral Doppler measurements. ICA peak systolic velocity (PSV) was the principal hemodynamic parameter, with ICA end-diastolic velocity (EDV) and the ICA-to-CCA PSV ratio used as supporting parameters. Stenosis <50% was generally characterized by ICA PSV <125 cm/s with <50% luminal narrowing; 50%–69% stenosis by ICA PSV of 125–230 cm/s with visible plaque and ≥50% luminal narrowing, with an ICA/CCA PSV ratio of approximately 2.0–4.0 and EDV of 40–100 cm/s as supportive findings; and ≥70% stenosis by ICA PSV >230 cm/s with substantial luminal narrowing, with an ICA/CCA PSV ratio >4.0 and EDV >100 cm/s as supportive findings. Near-occlusive lesions with residual flow were included in the 70%–99% category and interpreted using combined gray-scale, color Doppler, and spectral Doppler findings because velocities may be variable. Complete occlusion required absence of a patent lumen and detectable flow. Normal arteries and stenosis <50% were combined in the <50% category for analysis.

PSV, EDV, and the ICA/CCA PSV ratio were routinely acquired as part of stenosis assessment, but exact numeric velocity measurements were not consistently retrievable in structured form across the historical cohort; therefore, the present analyses used the final stenosis category documented in the index duplex report rather than retrospective modeling of raw Doppler velocities.

For sonographic quality assessment, a random 20% sample with available archived images was independently reassessed by a second trained reader who was unaware of subsequent outcome status. Segment-level plaque status, the simplified plaque score, maximal plaque thickness, total plaque number, and the presence of carotid stenosis ≥50% were reassessed using the available archived gray-scale, color Doppler, and spectral Doppler data. Interobserver agreement was evaluated using Cohen *κ* for categorical assessments and intraclass correlation coefficients for continuous ultrasound measurements.

The 12 prespecified predictors for the primary model were age, sex, BMI, systolic blood pressure, current smoking, diabetes mellitus, antihypertensive medication use, baseline statin use, low-density lipoprotein cholesterol (LDL-C), high-density lipoprotein cholesterol (HDL-C), eGFR, and the simplified carotid plaque score. Current smoking status was determined from the most recent structured smoking history or clinician documentation available on or before the index date. Diabetes mellitus was defined by a documented clinical diagnosis or ongoing glucose-lowering therapy. Baseline antihypertensive and statin use were determined from active medication records or prescriptions documented within 90 days before or on the index date. Atrial fibrillation (AF) was additionally recorded for baseline characterization and sensitivity analysis. Total cholesterol, triglycerides, fasting blood glucose, glycated hemoglobin (HbA1c), maximal plaque thickness, and total plaque number were additionally extracted when available for descriptive and supplementary analyses.

The primary outcome was the first occurrence of hard MACE within 5 years after the index date, defined as cardiovascular death, non-fatal myocardial infarction, or non-fatal ischemic stroke. Candidate non-fatal events were identified from longitudinal clinical records and diagnostic codes available within the institutional clinical information systems. When medical care received elsewhere was subsequently documented in accessible referral notes, discharge summaries, imaging reports, laboratory records, or other source documentation, these records were also reviewed during event adjudication. Cardiovascular deaths were additionally ascertained through linkage with the regional mortality registry. A comprehensive cross-hospital registry for non-fatal myocardial infarction or ischemic stroke was not available; therefore, a non-fatal event treated entirely outside the accessible healthcare system could only be identified if it was subsequently documented in an available clinical record. For patients without an observed primary event, follow-up was censored at the last date on which outcome status could be reliably ascertained from an accessible clinical record or registry source. Non-fatal myocardial infarction was adjudicated and classified by type according to the Fourth Universal Definition of Myocardial Infarction (13). The prespecified hard-MACE definition did not exclude type 2 or procedure-related myocardial infarction; therefore, these events were included when the diagnostic criteria for myocardial infarction were fulfilled. Type 3 myocardial infarction, which is fatal by definition, was not classified as a non-fatal myocardial infarction and was considered within cardiovascular death when applicable. Ischemic stroke was adjudicated on the basis of acute focal neurological dysfunction attributed to cerebral infarction, with computed tomography (CT) or magnetic resonance imaging (MRI) used to exclude intracranial hemorrhage and support the ischemic diagnosis (14, 15). Cardiovascular death was classified from death certification and available clinical records according to standardized cardiovascular endpoint definitions (15).

Secondary outcomes included expanded MACE, defined as hard MACE plus coronary or carotid revascularization, the individual components of the primary outcome, and all-cause mortality. Follow-up commenced on the index date. For the primary analysis, patients were followed until the first occurrence of hard MACE, death, the last date on which outcome status could be reliably ascertained, 5 years after the index date, or December 31, 2025, whichever occurred first. Secondary time-to-event outcomes were analyzed using the corresponding endpoint-specific follow-up, with patients censored at the first occurrence of the relevant endpoint, death when applicable, the last date of reliable outcome ascertainment, 5 years after the index date, or December 31, 2025. Primary outcome events were independently adjudicated by two investigators blinded to baseline carotid plaque scores, with disagreements resolved by a third senior adjudicator.

### 2.4 Sample size calculation

The minimum sample size for development of the time-to-event prediction model was estimated according to the framework proposed by Riley and colleagues (16, 17). Based on previous epidemiological data for populations without prior cardiovascular events (11, 18), a 5-year MACE risk of 4.5% was assumed for sample size planning.

The primary model included 12 prespecified predictor parameters. A 5-year cumulative MACE risk of 4.5% corresponded to an annual event rate of approximately 0.0092 per person-year. A prediction horizon of 5 years and a mean effective follow-up of 4.5 years were specified. In the absence of directly applicable estimates of model R^2^, an anticipated Nagelkerke R^2^ of 0.15 was used for planning, corresponding to a Cox–Snell R^2^ of approximately 0.044 under the specified event rate and follow-up assumptions (16, 17, 19). With a target global shrinkage factor of 0.90, calculation using the pmsampsize package yielded a minimum required sample size of approximately 2,394 analyzable patients. The target sample size was therefore set at approximately 2,400 patients, with approximately 100 primary outcome events expected. Allowing for a 10% exclusion rate due to missing core data or loss to follow-up, approximately 2,650–2,700 patient records were expected to require screening.

### 2.5 Statistical analysis

Continuous variables were summarized as mean ± standard deviation (SD) or median (interquartile range, IQR), as appropriate, and categorical variables as counts and percentages. Baseline characteristics were described overall and by primary MACE status. Examination indication, referral source, encounter setting, and selected cardiovascular risk factors were additionally summarized by ultrasound indication; these comparisons were descriptive and were not used for predictor selection. Follow-up duration was estimated using the reverse Kaplan–Meier method. Outcome-ascertainment completeness was defined as MACE or death during follow-up, or an accessible clinical record extending to within 12 months of the individual 5-year horizon. Event numbers, incidence rates per 1,000 person-years, and 5-year cumulative incidence were reported. Cumulative incidence functions were displayed across plaque-score categories of 0–1, 2–4, and 5–6, with non-cardiovascular death treated as a competing event. Plaque score was also cross-tabulated descriptively against maximal carotid stenosis categories (<50%, 50%–69%, 70%–99%, and occlusion), without hypothesis testing.

Missingness was summarized for all variables. The simplified plaque score required evaluable data for all six carotid segments and was not imputed. Missing values in other prespecified predictors were handled using multiple imputation by chained equations with 20 datasets; the imputation model included all predictors, the event indicator, the Nelson–Aalen cumulative hazard estimate, and relevant auxiliary variables. Outcomes were not imputed. In the primary Cox analysis, non-cardiovascular deaths were censored at death and other event-free patients at the last date of reliable outcome ascertainment.

Three models were prespecified. Model 1 included age, sex, BMI, systolic blood pressure, current smoking, diabetes, antihypertensive medication use, LDL-C, HDL-C, eGFR, and baseline statin use. Model 2 added binary carotid plaque presence, and Model 3 added the 0–6 plaque score to Model 1 and constituted the primary model. Multivariable Cox regression was used for model development. Continuous predictors were retained on their original scales and modeled linearly. Proportional hazards assumptions were assessed using scaled Schoenfeld residuals and diagnostic plots.

Discrimination was evaluated using Harrell C-indices and time-dependent AUCs at 3 and 5 years, overall prediction error using the 5-year Brier score, and calibration using the calibration slope and predicted-versus-observed 5-year risk plots. Incremental performance across Models 1–3 was assessed using differences in C-index, 3-year and 5-year AUCs, and 5-year Brier score. Paired comparisons used 1,000 patient-level bootstrap resamples, with both models evaluated within the same resample. Bootstrap standard errors were derived from the distribution of paired differences, and two-sided *P* values from the corresponding bootstrap z statistics. Brier-score differences were expressed as reductions in prediction error. Model 3 versus Models 1 and 2 were the primary comparisons; Model 2 versus Model 1 was secondary. Decision curve analysis compared net benefit across prespecified 5-year risk thresholds of 1%–10%.

Internal validation used 1,000 patient-level bootstrap resamples incorporating the prespecified missing-data and modeling procedures. Apparent and optimism-corrected C-indices, Brier scores, and calibration slopes were obtained and pooled across imputed datasets as appropriate. Final pooled coefficients, the 5-year baseline survival estimate, and the individual risk equation were reported.

Sensitivity analyses excluded MACE within 90 days, restricted analyses to patients with complete archived PACS images, excluded baseline carotid stenosis ≥50% or AF, applied Fine–Gray competing-risk regression, used complete cases, and substituted expanded MACE as the outcome. Additional analyses restricted the myocardial infarction component to type 1 MI and restricted the cohort to patients with high-continuity follow-up, defined by at least one accessible clinical encounter during each 18-month interval until MACE, death, or 5 years.

To assess potential modification across the COVID-19 transition, follow-up was split at March 11, 2020. An extended Cox model in counting-process form included a time-varying calendar-period indicator and plaque score   ×  calendar-period interaction, and was repeated using the type 1 MI–restricted endpoint. As a complementary frozen-model analysis, 3-year predictions from Model 3 were evaluated without coefficient or baseline-survival re-estimation in nonoverlapping cohorts whose 3-year horizons occurred entirely before or after March 11, 2020. The pre-pandemic cohort included index examinations from January 1, 2015, through March 10, 2017, and the COVID-19-era cohort examinations from March 11 through December 31, 2020. Three-year AUC, Brier score, calibration-in-the-large, and calibration slope were evaluated; these analyses were exploratory. Baseline characteristics were also compared between the analyzed cohort and patients excluded for missing core data or incomplete follow-up.

Prespecified exploratory subgroup analyses were performed by sex and diabetes status using the frozen final model without predictor reselection or coefficient refitting. Subgroup C-indices with 95% CIs were reported, and interaction terms assessed potential heterogeneity in the plaque-score association. All tests were two-sided, with *P* < 0.05 considered statistically significant.

Analyses were performed in R version 4.5.1 (R Foundation for Statistical Computing, Vienna, Austria) using the mice, survival, riskRegression, timeROC, cmprsk, dcurves, and pmsampsize packages.

## 3 Results

### 3.1 Study selection and baseline characteristics

A total of 4,218 bilateral carotid ultrasound examinations were identified, representing 3,238 unique patients after patient-level deduplication. After exclusion of patients aged <40 years (*n* = 173), those with prior clinical atherosclerotic CVD or revascularization (*n* = 341), examinations performed for acute or suspected acute cardiovascular or cerebrovascular events (*n* = 112), incomplete six-segment plaque assessment (*n* = 128), and unavailable post-index outcome ascertainment (*n* = 71), 2,413 patients were included in the analytic cohort (Figure 1). The primary indication for carotid ultrasonography was routine health evaluation in 590 patients (24.5%), cardiovascular risk assessment in 1,078 (44.7%), and stable chronic disease management in 745 (30.9%). Overall, 1,681 patients (69.7%) underwent the index examination as outpatients and 732 (30.3%) as inpatients. The ordering service was the health management/physical examination center for 590 patients (24.5%), cardiovascular medicine for 883 (36.6%), cerebrovascular/neurology services for 540 (22.4%), and other internal medicine services for 400 (16.6%). Cardiovascular risk burden increased across the three indication groups; patients undergoing ultrasonography during stable chronic disease management were older and more frequently had diabetes and preventive medication use than those undergoing routine health evaluation. Carotid plaque was present in 41.4%, 66.1%, and 80.9% of patients in the routine health evaluation, cardiovascular risk assessment, and stable chronic disease management groups, respectively (Supplementary Table S1). During a median follow-up of 4.92 years (IQR, 4.31–5.00; 10,614.8 person-years), 108 patients (4.5%) experienced primary MACE. Overall, 2,204 patients (91.3%) either experienced MACE or death or had an accessible clinical record extending to within 12 months of their 5-year follow-up horizon. The remaining participants were censored at the last date on which outcome status could be reliably ascertained. Missingness among prespecified predictors was low, ranging from 1.2% for systolic blood pressure to 4.4% for LDL-C and HDL-C; the simplified carotid plaque score was complete in all included patients. Patients with MACE were older, had higher systolic blood pressure, and more frequently had diabetes, current smoking, and AF, together with lower eGFR and greater carotid plaque burden (all *P* ≤ 0.002) (Table 1). Baseline characteristics of the analytic cohort and patients excluded because of incomplete plaque assessment or unavailable outcome ascertainment are summarized in Supplementary Table S2.

**Figure 1:**
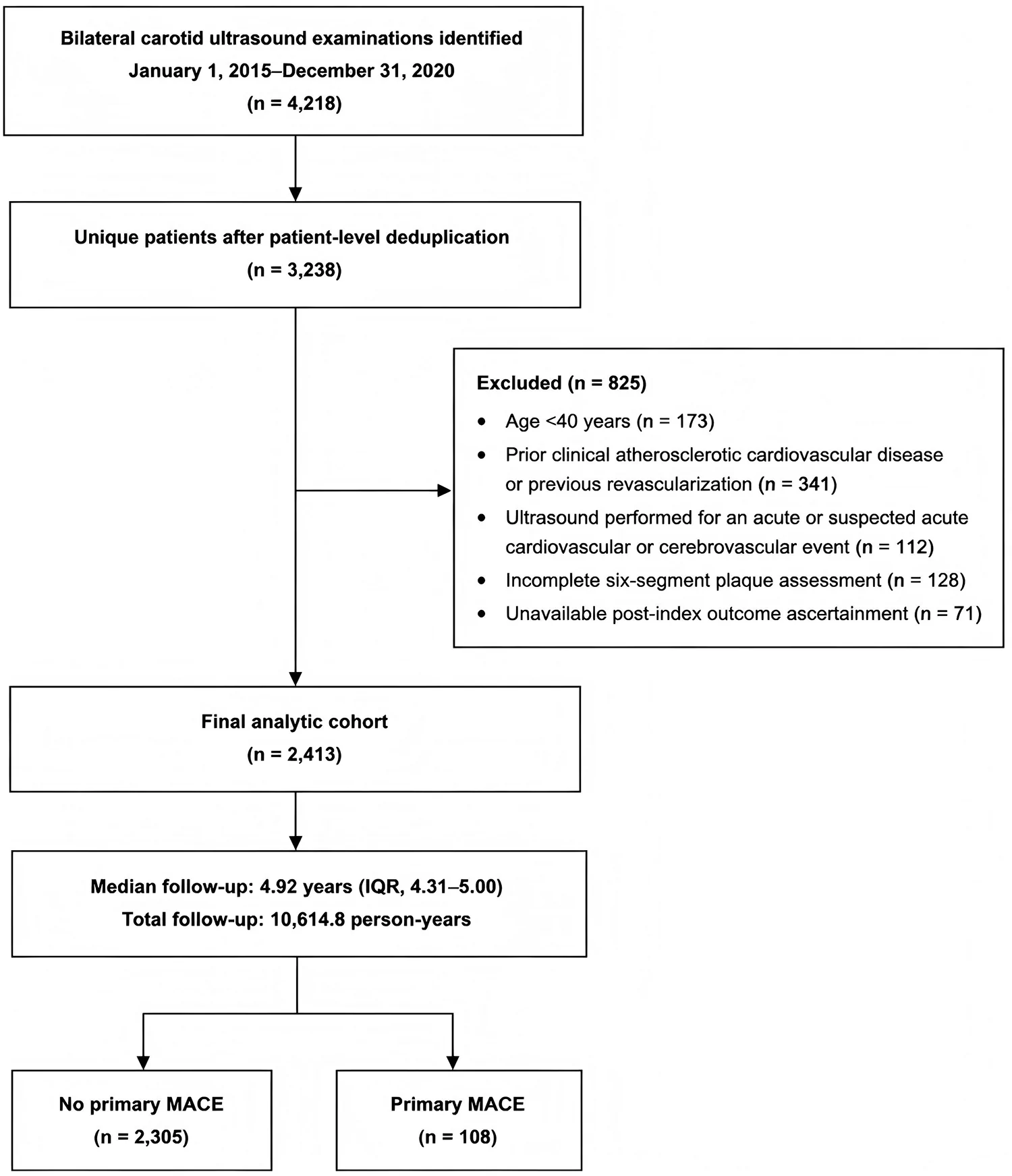
Flow diagram yyof patient selection and follow-up.

**Table 1:** Baseline clinical, laboratory, and carotid ultrasound characteristics of the study cohort.

Variable	Overall (*N* = 2,413)	No MACE (*n* = 2,305)	MACE (*n* = 108)	Statistic	*P* value
Age, years	59.8 ± 9.7	59.5 ± 9.6	66.4 ± 9.4	t = −7.34	<0.001
Male sex	1,237 (51.3)	1,167 (50.6)	70 (64.8)	*χ*^2^ = 7.75	0.005
BMI, kg/m^2^	24.8 ± 3.2	24.8 ± 3.1	25.7 ± 3.5	t = −2.55	0.012
Systolic blood pressure, mmHg	130.9 ± 15.8	130.4 ± 15.6	139.6 ± 16.7	t = −5.58	<0.001
Current smoking	379 (15.7)	349 (15.1)	30 (27.8)	χ^2^ = 11.51	<0.001
Diabetes mellitus	362 (15.0)	334 (14.5)	28 (25.9)	χ^2^ = 9.70	0.002
Antihypertensive medication use	769 (31.9)	725 (31.5)	44 (40.7)	χ^2^ = 3.68	0.055
Baseline statin use	641 (26.6)	608 (26.4)	33 (30.6)	χ^2^ = 0.72	0.396
LDL-C, mmol/L	2.93 ± 0.76	2.92 ± 0.76	3.14 ± 0.74	t = −3.00	0.003
HDL-C, mmol/L	1.28 ± 0.31	1.29 ± 0.31	1.15 ± 0.27	t = 5.03	<0.001
eGFR, mL/min/1.73 m^2^	87.5 ± 17.5	88.0 ± 17.3	77.6 ± 18.8	t = 5.48	<0.001
AF	56 (2.3)	47 (2.0)	9 (8.3)	χ^2^ = 15.36	<0.001
Total cholesterol, mmol/L	4.74 ± 0.92	4.74 ± 0.93	4.83 ± 0.79	t = −1.15	0.254
Triglycerides, mmol/L	1.42 (1.04–1.94)	1.42 (1.03–1.92)	1.64 (1.19–2.27)	Z = −2.63	0.009
Fasting blood glucose, mmol/L	5.36 (4.84–6.01)	5.35 (4.83–5.97)	6.03 (5.16–7.08)	Z = −5.49	<0.001
HbA1c, %	5.81 (5.43–6.25)	5.80 (5.42–6.21)	6.40 (5.81–6.90)	Z = −6.26	<0.001
Any carotid plaque	1,560 (64.6)	1,462 (63.4)	98 (90.7)	χ^2^ = 32.49	<0.001
Bilateral carotid plaque	650 (26.9)	585 (25.4)	65 (60.2)	χ^2^ = 61.75	<0.001
Simplified carotid plaque score	1 (0–3)	1 (0–3)	3 (2–5)	Z = −9.39	<0.001
Maximal plaque thickness, mm^1^	1.95 (1.74–2.20)	1.94 (1.74–2.18)	2.14 (1.87–2.39)	Z = −4.27	<0.001
Total plaque number	1 (0–3)	1 (0–3)	4 (2–5)	Z = −8.06	<0.001
Maximal carotid stenosis ≥50%	122 (5.1)	106 (4.6)	16 (14.8)	χ^2^ = 20.35	<0.001

Values are presented as mean ± SD, median (IQR), or *n* (%), as appropriate. Continuous normally distributed variables were compared using independent-samples t tests, skewed continuous variables using Mann–Whitney U tests with standardized Z statistics, and categorical variables using χ^2^ tests. *P* values were descriptive and were not used for predictor selection. Missing values were present for BMI in 62 patients (2.6%), systolic blood pressure in 30 (1.2%), LDL-C in 107 (4.4%), HDL-C in 107 (4.4%), eGFR in 49 (2.0%), total cholesterol in 132 (5.5%), triglycerides in 150 (6.2%), fasting blood glucose in 207 (8.6%), and HbA1c in 890 (36.9%). ^1^Maximal plaque thickness was summarized only among patients with detectable carotid plaque: overall, *n* = 1,560; no MACE, *n* = 1,462; and MACE, *n* = 98.

### 3.2 Carotid plaque distribution, simplified plaque score, and interobserver reliability

Carotid plaque was detected in 1,560 patients (64.6%), including unilateral plaque in 910 (37.7%) and bilateral plaque in 650 (26.9%). Plaque was most frequently detected at the carotid bulbs, with lower prevalence in the ICAs and CCAs (Table 2). The simplified carotid plaque score had a median of 1 (IQR, 0–3), with 853 patients (35.4%) having a score of 0; the full 0–6 score distribution is shown in Figure 2. Among plaque-positive patients, the median maximal plaque thickness was 1.95 mm (IQR, 1.74–2.20) and the median total plaque number was 2 (IQR, 1–4). Maximal carotid stenosis ≥50% was present in 122 patients (5.1%), including 98 with 50%–69% stenosis, 21 with 70%–99% stenosis, and 3 with occlusion (Table 2).

**Table 2:** Baseline carotid ultrasound characteristics and distribution of the simplified carotid plaque score.

Characteristic	Overall (*N* = 2,413)
Any carotid plaque	1,560 (64.6)
No carotid plaque	853 (35.4)
Unilateral carotid plaque	910 (37.7)
Bilateral carotid plaque	650 (26.9)
Segment-level plaque distribution	
Left CCA	626 (25.9)
Right CCA	610 (25.3)
Left carotid bulb	871 (36.1)
Right carotid bulb	842 (34.9)
Left ICA	716 (29.7)
Right ICA	694 (28.8)
Simplified carotid plaque score	
Median (IQR)	1 (0–3)
Score 0	853 (35.4)
Score 1	411 (17.0)
Score 2	337 (14.0)
Score 3	318 (13.2)
Score 4	245 (10.2)
Score 5	154 (6.4)
Score 6	95 (3.9)
Additional plaque characteristics	
Total plaque number, median (IQR), overall cohort	1 (0–3)
Total plaque number, median (IQR), plaque-positive patients	2 (1–4)
Maximal plaque thickness, mm, median (IQR)^1^	1.95 (1.74–2.20)
Maximal carotid stenosis severity	
<50%	2,291 (94.9)
50%–69%	98 (4.1)
70%–99%	21 (0.9)
Occlusion	3 (0.1)
Maximal carotid stenosis ≥50%	122 (5.1)

Values are presented as *n* (%) or median (IQR), as appropriate. Segment-level percentages represent the proportion of the entire analytic cohort with plaque detected in the specified arterial segment. Unilateral plaque indicates plaque confined to either the left or right carotid arterial territory, whereas bilateral plaque indicates plaque detected on both sides. The simplified carotid plaque score represents the number of plaque-positive segments across the bilateral CCAs, carotid bulbs, and proximal ICAs and ranges from 0 to 6. ^1^Maximal plaque thickness was summarized among the 1,560 patients with detectable carotid plaque. Percentages may not total 100% because of rounding.

**Figure 2:**
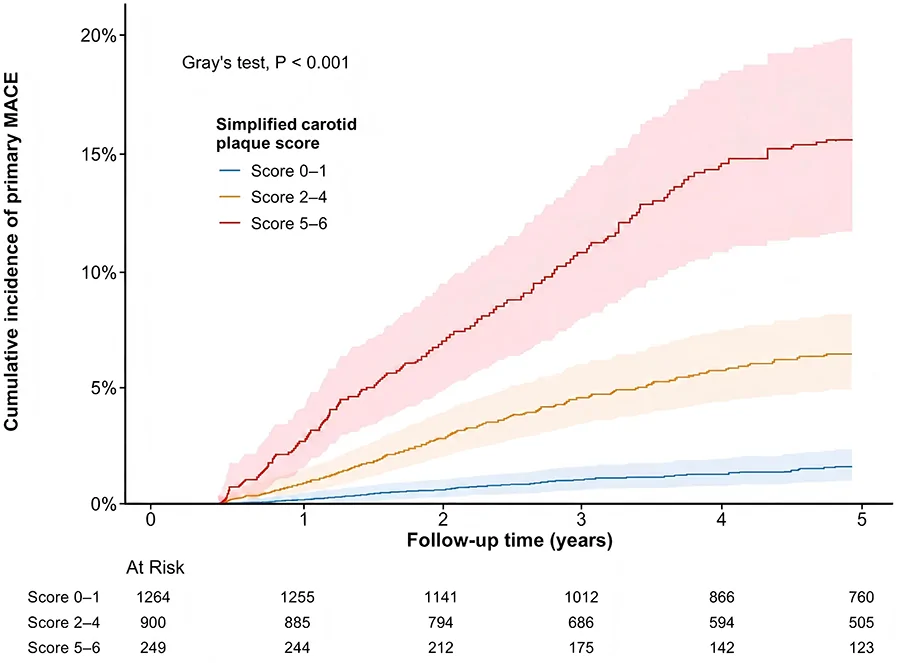
Distribution of the simplified carotid plaque score. The figure displays the distribution of simplified carotid plaque scores from 0 to 6 among the 2,413 patients in the analytic cohort. The horizontal axis represents the plaque score and the vertical axis represents the number of patients. Bars correspond to individual score categories, with patient counts and percentages displayed above each bar.

Cross-tabulation demonstrated that plaque extent and stenosis severity were related but distinct ultrasound phenotypes (Supplementary Table S3). The proportion with maximal stenosis ≥50% increased from 0% among patients with a plaque score of 0 to 5.6% for score 1, 5.6% for score 2, 7.5% for score 3, 9.8% for score 4, 11.7% for score 5, and 14.7% for score 6. Nevertheless, among the 249 patients with plaque scores of 5–6, 217 (87.1%) still had maximal stenosis <50%, indicating that a high plaque score predominantly represented diffuse multisegment plaque rather than hemodynamically important carotid stenosis.

In the 483-patient image reassessment sample, *κ* values for segment-level plaque classification ranged from 0.84 to 0.89, while agreement for any plaque and bilateral plaque was 0.91 and 0.90, respectively. Agreement for maximal carotid stenosis ≥50% was also high (*κ* = 0.88; 95% CI, 0.80–0.95). The ICC was 0.94 (95% CI, 0.93–0.95) for the simplified plaque score, 0.92 (95% CI, 0.90–0.94) for maximal plaque thickness, and 0.91 (95% CI, 0.89–0.93) for total plaque number (Supplementary Table S4).

### 3.3 Five-year primary and secondary cardiovascular and cerebrovascular outcomes

During 10,614.8 person-years of follow-up for the primary outcome, 108 patients experienced a first primary MACE, corresponding to an incidence rate of 10.2 per 1,000 person-years (95% CI, 8.3–12.3) and a 5-year cumulative incidence of 4.7% (95% CI, 3.9–5.6). Among these first primary events, 24 (22.2%) were cardiovascular deaths, 30 (27.8%) were non-fatal myocardial infarctions, and 54 (50.0%) were non-fatal ischemic strokes. Of the 30 non-fatal myocardial infarctions constituting the first hard-MACE event, 25 (83.3%) were classified as type 1, 4 (13.3%) as type 2, and 1 (3.3%) as type 4a myocardial infarction. When the individual components were evaluated as separate secondary outcomes over their respective follow-up periods, cardiovascular death occurred in 24 patients, non-fatal myocardial infarction in 35, and non-fatal ischemic stroke in 60, with 5-year cumulative incidences of 1.0% (95% CI, 0.7–1.5), 1.5% (95% CI, 1.0–2.1), and 2.6% (95% CI, 2.0–3.4), respectively. Among the 35 patients with at least one adjudicated non-fatal myocardial infarction during follow-up, the first myocardial infarction was classified as type 1 in 29 patients (82.9%), type 2 in 5 (14.3%), and type 4a in 1 (2.9%); no type 4b, type 4c, or type 5 myocardial infarctions were identified. Expanded MACE occurred in 153 patients, corresponding to a 5-year cumulative incidence of 6.6% (95% CI, 5.6–7.7). Coronary and carotid revascularization occurred in 41 and 12 patients, respectively. A total of 86 deaths were recorded during follow-up, comprising 24 cardiovascular and 62 non-cardiovascular deaths; the 5-year cumulative all-cause mortality was 3.8% (95% CI, 3.1–4.7) (Table 3). Among 1,264 patients with simplified carotid plaque scores of 0–1, 18 experienced primary MACE, with a 5-year cumulative incidence of 1.5% (95% CI, 0.9–2.3). Corresponding values were 53 events and 6.1% (95% CI, 4.8–7.8) among 900 patients with scores of 2–4 and 37 events and 15.4% (95% CI, 11.3–20.4) among 249 patients with scores of 5–6. The cumulative incidence functions differed across the three plaque score categories (Gray test, *P* < 0.001) (Figure 3).

**Table 3:** Incidence and composition of primary and secondary cardiovascular and cerebrovascular outcomes.

Outcome	Events, *n* (%)	Incidence rate per 1,000 person-years (95% CI)	5-year cumulative incidence, % (95% CI)
Primary outcome			
Hard MACE	108 (4.5)	10.2 (8.3–12.3)	4.7 (3.9–5.6)
Composition of first hard MACE, *n* (% of first events)			
Cardiovascular death	24 (22.2)	—	—
Non-fatal myocardial infarction	30 (27.8)	—	—
└ Type 1 myocardial infarction, *n* (% of first-event MI)	25 (83.3)	—	—
└ Type 2 myocardial infarction, *n* (% of first-event MI)	4 (13.3)	—	—
└ Type 4a myocardial infarction, *n* (% of first-event MI)	1 (3.3)	—	—
Non-fatal ischemic stroke	54 (50.0)	—	—
Secondary outcomes			
Expanded MACE	153 (6.3)	14.6 (12.4–17.1)	6.6 (5.6–7.7)
Cardiovascular death	24 (1.0)	2.2 (1.4–3.3)	1.0 (0.7–1.5)
Non-fatal myocardial infarction	35 (1.5)	3.3 (2.3–4.6)	1.5 (1.0–2.1)
└ Type 1 myocardial infarction, *n* (% of MI cases)	29 (82.9)	—	—
└ Type 2 myocardial infarction, *n* (% of MI cases)	5 (14.3)	—	—
└ Type 4a myocardial infarction, *n* (% of MI cases)	1 (2.9)	—	—
Non-fatal ischemic stroke	60 (2.5)	5.6 (4.3–7.2)	2.6 (2.0–3.4)
Coronary revascularization	41 (1.7)	3.8 (2.7–5.2)	1.8 (1.3–2.4)
Carotid revascularization	12 (0.5)	1.1 (0.6–1.9)	0.5 (0.3–0.9)
All-cause mortality	86 (3.6)	8.1 (6.5–10.0)	3.8 (3.1–4.7)
Primary MACE according to simplified carotid plaque score			
Score 0–1 (*n* = 1,264)	18 (1.4)	—	1.5 (0.9–2.3)
Score 2–4 (*n* = 900)	53 (5.9)	—	6.1 (4.8–7.8)
Score 5–6 (*n* = 249)	37 (14.9)	—	15.4 (11.3–20.4)

Event percentages for the primary and secondary outcomes are calculated using the corresponding cohort denominator. Percentages for the composition of first hard MACE are calculated among the 108 patients with a first primary event; cardiovascular death, non-fatal myocardial infarction, and non-fatal ischemic stroke are mutually exclusive as first events and sum to 108. Myocardial infarction subtypes were adjudicated according to the Fourth Universal Definition of Myocardial Infarction. Percentages for myocardial infarction subtypes under the first hard-MACE composition are calculated among the 30 myocardial infarctions constituting the first primary event. Percentages for myocardial infarction subtypes under the secondary outcome are calculated among the 35 patients with at least one adjudicated non-fatal myocardial infarction and refer to the first qualifying myocardial infarction in each patient. Individual components of hard MACE were additionally analyzed as separate secondary time-to-event outcomes and therefore were not mutually exclusive during follow-up. Expanded MACE was defined by the first occurrence of hard MACE, coronary revascularization, or carotid revascularization. Incidence rates are expressed per 1,000 person-years; the primary-outcome incidence rate was based on 10,614.8 person-years, whereas secondary-outcome incidence rates used endpoint-specific person-time. For hard MACE and expanded MACE, non-cardiovascular death was treated as a competing event when estimating 5-year cumulative incidence. For non-fatal myocardial infarction, non-fatal ischemic stroke, and revascularization outcomes, death before the corresponding event was treated as a competing event. For cardiovascular death, non-cardiovascular death was treated as the competing event. All-cause mortality was estimated without competing-risk adjustment. Plaque score categories were used only for descriptive visualization; the original 0–6 score was retained for prediction modeling. CI, confidence interval; MACE, major adverse cardiovascular and cerebrovascular events; MI, myocardial infarction.

**Figure 3:**
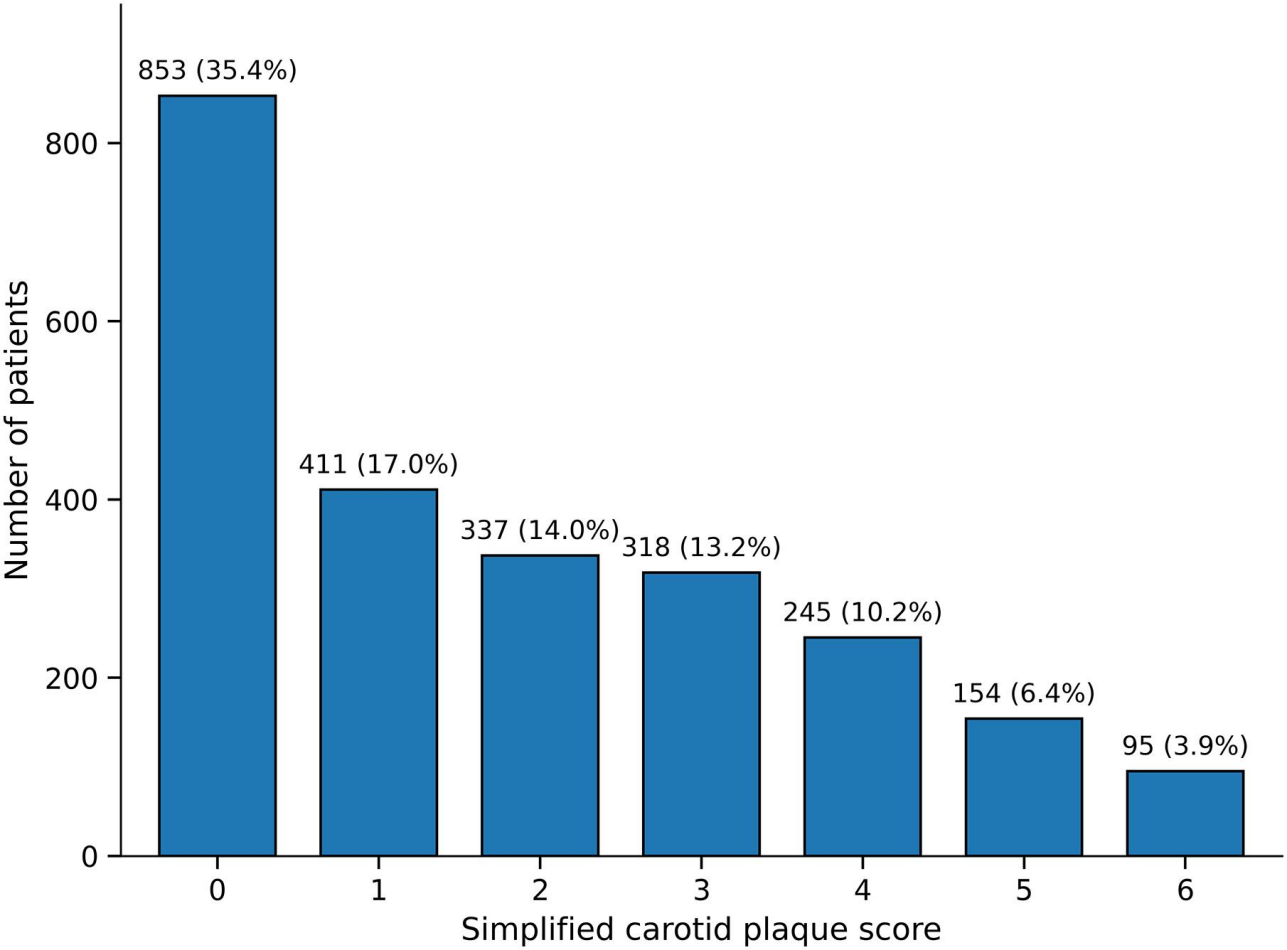
Cumulative incidence of MACE according to simplified carotid plaque score categories. The figure displays cumulative incidence functions for primary MACE according to simplified carotid plaque score categories of 0–1, 2–4, and 5–6 points over 5 years after the index date, with non-cardiovascular death treated as a competing event. The horizontal axis represents follow-up time in years, and the vertical axis represents cumulative incidence of primary MACE. Shaded bands denote 95% CIs, and numbers at risk are displayed below the horizontal axis. The between-group comparison is based on Gray's test.

### 3.4 Prediction model development

All prespecified predictors were retained in the three Cox models without selection according to individual *P* values. In Model 1, age, systolic blood pressure, current smoking, diabetes, and eGFR were associated with primary MACE. After the presence of any carotid plaque was added in Model 2, any carotid plaque was associated with a higher hazard of MACE (HR, 2.21; 95% CI, 1.40–3.49; *P* < 0.001), while the estimates for the clinical predictors changed modestly. In Model 3, the binary plaque variable was replaced by the 0–6 simplified carotid plaque score. Each 1-point increase in the simplified carotid plaque score was associated with an HR of 1.32 (95% CI, 1.21–1.44; *P* < 0.001). Age (HR per year, 1.043; 95% CI, 1.026–1.060; *P* < 0.001), systolic blood pressure (HR per mmHg, 1.011; 95% CI, 1.004–1.018; *P* = 0.002), current smoking (HR, 1.47; 95% CI, 1.02–2.12; *P* = 0.039), and eGFR (HR per mL/min/1.73 m^2^, 0.991; 95% CI, 0.983–0.999; *P* = 0.029) were associated with primary MACE in Model 3. The remaining prespecified predictors were retained in the model irrespective of statistical significance (Table 4). The pooled 5-year baseline survival estimate for Model 3 was 0.99938, and the coefficients and equation required to calculate individual 5-year MACE risk are provided in the notes to Table 4.

**Table 4:** Predictors and model parameters of the three prediction models.

Predictor	Model 1 HR (95% CI)	*P* value	Model 2 HR (95% CI)	*P* value	Model 3 *β*	Model 3 HR (95% CI)	*P* value
Age, per year	1.048 (1.031–1.065)	<0.001	1.046 (1.029–1.063)	<0.001	0.0421	1.043 (1.026–1.060)	<0.001
Male sex	1.34 (0.93–1.94)	0.119	1.29 (0.89–1.87)	0.179	0.2231	1.25 (0.86–1.82)	0.242
BMI, per kg/m^2^	1.029 (0.971–1.090)	0.337	1.027 (0.968–1.087)	0.389	0.0218	1.022 (0.966–1.081)	0.455
Systolic blood pressure, per mmHg	1.013 (1.006–1.020)	<0.001	1.012 (1.005–1.019)	0.001	0.0109	1.011 (1.004–1.018)	0.002
Current smoking	1.57 (1.10–2.24)	0.013	1.50 (1.05–2.15)	0.026	0.3853	1.47 (1.02–2.12)	0.039
Diabetes mellitus	1.49 (1.04–2.14)	0.031	1.44 (1.00–2.07)	0.051	0.3436	1.41 (0.97–2.05)	0.071
Antihypertensive medication use	1.17 (0.82–1.67)	0.391	1.15 (0.80–1.64)	0.451	0.1133	1.12 (0.78–1.60)	0.540
Baseline statin use	0.94 (0.66–1.35)	0.745	0.92 (0.64–1.32)	0.650	−0.0943	0.91 (0.63–1.31)	0.612
LDL-C, per mmol/L	1.20 (0.97–1.49)	0.091	1.18 (0.95–1.47)	0.132	0.1484	1.16 (0.93–1.45)	0.190
HDL-C, per mmol/L	0.69 (0.39–1.24)	0.215	0.71 (0.40–1.28)	0.258	−0.3285	0.72 (0.40–1.30)	0.280
eGFR, per mL/min/1.73 m^2^	0.988 (0.980–0.997)	0.008	0.989 (0.980–0.998)	0.015	−0.0090	0.991 (0.983–0.999)	0.029
Any carotid plaque	—	—	2.21 (1.40–3.49)	<0.001	—	—	—
Simplified carotid plaque score, per point	—	—	—	—	0.2776	1.32 (1.21–1.44)	<0.001

Estimates were pooled across the 20 imputed datasets. HRs for continuous predictors represent the relative hazard associated with a 1-unit increase in the corresponding variable. HRs for age, BMI, systolic blood pressure, and eGFR are displayed to three decimal places to avoid loss of information from rounding of estimates close to 1. Binary variables were coded as 1 for presence and 0 for absence; male sex was coded as 1 and female sex as 0. Model 1 included the 11 prespecified clinical predictors. Model 2 included the Model 1 predictors plus the presence of any carotid plaque. Model 3 included the Model 1 predictors plus the 0–6 simplified carotid plaque score and constituted the final primary model. All prespecified predictors were retained regardless of individual *P* values. For Model 3, the linear predictor was calculated as: LP = 0.0421 × age + 0.2231 × male sex + 0.0218 × BMI + 0.0109 × systolic blood pressure + 0.3853 × current smoking + 0.3436 × diabetes mellitus + 0.1133 × antihypertensive medication use−0.0943 × baseline statin use + 0.1484 × LDL-C−0.3285 × HDL-C−0.0090 × eGFR + 0.2776 × simplified carotid plaque score. The pooled 5-year baseline survival estimate was 0.99938. Individual 5-year MACE risk was calculated as: 5-year MACE risk = 1−0.99938^exp(LP). Continuous predictors are entered using the units shown in the table. CI, confidence interval; HR, hazard ratio; LP, linear predictor.

### 3.5 Incremental predictive value of the simplified carotid plaque score

Model performance improved sequentially after incorporation of carotid plaque information (Table 5). The apparent Harrell C-index increased from 0.753 (95% CI, 0.709–0.797) in Model 1 to 0.776 (95% CI, 0.735–0.817) in Model 2 and 0.804 (95% CI, 0.765–0.843) in Model 3. Compared with Model 1, Model 3 increased the C-index by 0.051 (95% CI, 0.032–0.070; *P* < 0.001), the 3-year AUC by 0.052 (95% CI, 0.030–0.074; *P* < 0.001), and the 5-year AUC by 0.051 (95% CI, 0.031–0.071; *P* < 0.001), while reducing the 5-year Brier score by 0.0036 (95% CI, 0.0019–0.0054; *P* < 0.001). Compared with Model 2, Model 3 increased the C-index by 0.028 (95% CI, 0.013–0.043; *P* < 0.001), the 3-year AUC by 0.028 (95% CI, 0.010–0.046; *P* = 0.002), and the 5-year AUC by 0.028 (95% CI, 0.011–0.045; *P* = 0.001), and reduced the 5-year Brier score by 0.0021 (95% CI, 0.0007–0.0035; *P* = 0.003). The secondary comparison of Model 2 versus Model 1 showed smaller improvements in C-index (*Δ* = 0.023; 95% CI, 0.008–0.039; *P* = 0.004), 3-year AUC (*Δ* = 0.024; 95% CI, 0.006–0.042; *P* = 0.009), 5-year AUC (*Δ* = 0.023; 95% CI, 0.007–0.040; *P* = 0.006), and 5-year Brier score (reductio*n* = 0.0015; 95% CI, 0.0003–0.0028; *P* = 0.019). Mean predicted 5-year risk was 4.8% in Model 3, matching the 4.8% event probability estimated under the primary Cox analysis framework. Apparent calibration slopes were approximately 1.00 for all three models.

**Table 5:** Discrimination, overall prediction error, calibration, and paired incremental comparisons of the three prediction models.

Performance measure	Model 1	Model 2	Model 3
Harrell C-index (95% CI)	0.753 (0.709–0.797)	0.776 (0.735–0.817)	0.804 (0.765–0.843)
3-year time-dependent AUC (95% CI)	0.758 (0.703–0.813)	0.782 (0.730–0.834)	0.810 (0.762–0.858)
5-year time-dependent AUC (95% CI)	0.756 (0.712–0.800)	0.779 (0.737–0.821)	0.807 (0.768–0.846)
5-year Brier score	0.0417	0.0402	0.0381
Mean predicted 5-year risk, %	4.7	4.8	4.8
Apparent calibration slope	1.00	1.00	1.00
Incremental performance vs Model 1			
*Δ*C-index (95% CI); *P* value	Reference	0.023 (0.008–0.039); *P* = 0.004	0.051 (0.032–0.070); *P* < 0.001
*Δ*3-year AUC (95% CI); *P* value	Reference	0.024 (0.006–0.042); *P* = 0.009	0.052 (0.030–0.074); *P* < 0.001
*Δ*5-year AUC (95% CI); *P* value	Reference	0.023 (0.007–0.040); *P* = 0.006	0.051 (0.031–0.071); *P* < 0.001
Reduction in 5-year Brier score (95% CI); *P* value	Reference	0.0015 (0.0003–0.0028); *P* = 0.019	0.0036 (0.0019–0.0054); *P* < 0.001
Model 3 vs. Model 2			
*Δ*C-index (95% CI); *P* value	—	—	0.028 (0.013–0.043); *P* < 0.001
*Δ*3-year AUC (95% CI); *P* value	—	—	0.028 (0.010–0.046); *P* = 0.002
*Δ*5-year AUC (95% CI); *P* value	—	—	0.028 (0.011–0.045); *P* = 0.001
Reduction in 5-year Brier score (95% CI); *P* value	—	—	0.0021 (0.0007–0.0035); *P* = 0.003

Model 1 included the 11 prespecified clinical predictors. Model 2 included the Model 1 predictors plus any carotid plaque, and Model 3 included the Model 1 predictors plus the 0–6 simplified carotid plaque score. Values represent apparent model performance before bootstrap optimism correction. Positive *Δ*C-index and *Δ*AUC values indicate higher discrimination relative to the reference model, whereas positive values for reduction in Brier score indicate lower prediction error. Differences in C-index, 3-year and 5-year time-dependent AUCs, and 5-year Brier score and their 95% CIs were estimated using 1,000 paired patient-level bootstrap resamples, with both models in each comparison evaluated within the same resample. Bootstrap standard errors were calculated from the distribution of resample-specific paired differences, and two-sided bootstrap-based *P* values were derived from the corresponding bootstrap z statistics. Model 3 versus Model 1 and Model 3 versus Model 2 were the primary incremental comparisons; Model 2 versus Model 1 was secondary. The 5-year event probability used for calibration assessment was estimated under the censoring framework corresponding to the primary Cox analysis. Apparent calibration slopes are expected to be approximately 1.00 when evaluated in the same data used for model development; optimism-corrected calibration is reported in Section 3.6. AUC, area under the receiver operating characteristic curve; CI, confidence interval.

### 3.6 Internal validation and clinical utility

After 1,000 bootstrap resamples, the optimism-corrected C-index was 0.744 for Model 1, 0.765 for Model 2, and 0.792 for Model 3; the corresponding 5-year Brier scores were 0.0424, 0.0411, and 0.0393, and calibration slopes were 0.95, 0.94, and 0.93, respectively (Supplementary Table S5). Comparative 3-year and 5-year time-dependent ROC curves for Models 1–3 are shown in Figures 4A,B respectively. The corresponding AUCs were 0.758, 0.782, and 0.810 at 3 years and 0.756, 0.779, and 0.807 at 5 years for Models 1, 2, and 3, respectively. Bootstrap optimism-corrected 5-year calibration curves for all three models are shown in Figure 4C. Calibration was broadly similar across models, with corrected slopes of 0.95, 0.94, and 0.93; for Model 3, modest overprediction remained at the upper end of predicted risk, with mean predicted and observed risks of 15.8% and 14.2% in the highest risk decile. In DCA, Model 3 showed greater net benefit than Models 1 and 2 across most thresholds from 2% to 10%. At the 5% threshold, net benefit was 0.024 for Model 3, 0.020 for Model 2, and 0.017 for Model 1 (Figure 4D).

**Figure 4:**
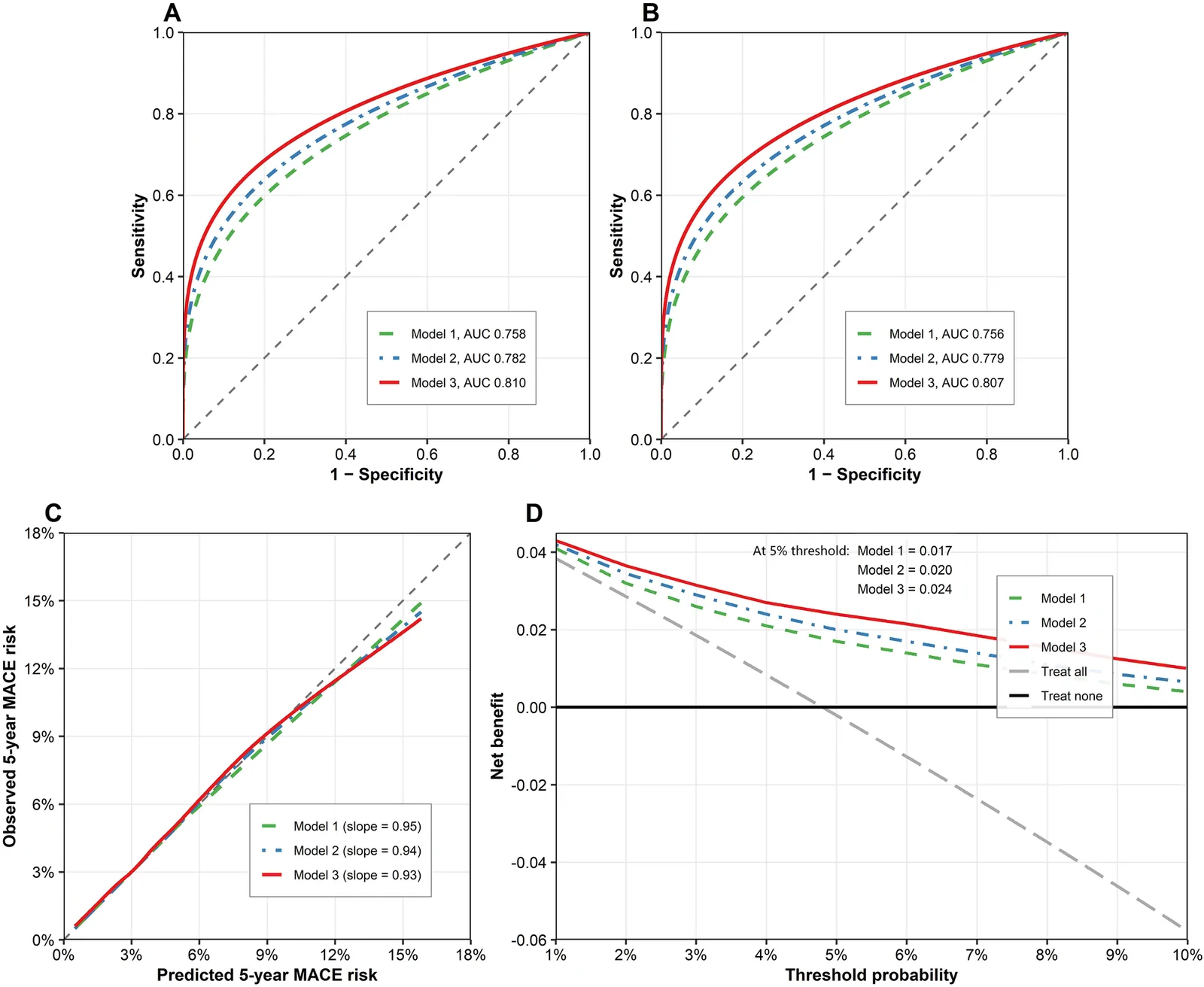
Comparative discrimination, calibration, and clinical net benefit of the three prediction models. **(A)** Apparent 3-year time-dependent receiver operating characteristic (ROC) curves for Models 1–3. The corresponding AUCs were 0.758 (95% CI, 0.703–0.813), 0.782 (95% CI, 0.730–0.834), and 0.810 (95% CI, 0.762–0.858), respectively. **(B)** Apparent 5-year time-dependent ROC curves for Models 1–3, with corresponding AUCs of 0.756 (95% CI, 0.712–0.800), 0.779 (95% CI, 0.737–0.821), and 0.807 (95% CI, 0.768–0.846), respectively. **(C)** Bootstrap optimism-corrected 5-year calibration curves for Models 1–3. The horizontal axis represents predicted 5-year MACE risk and the vertical axis represents observed 5-year risk estimated under the censoring framework used for the primary Cox analysis; the 45° line indicates perfect calibration. The optimism-corrected calibration slopes were 0.95, 0.94, and 0.93 for Models 1, 2, and 3, respectively. **(D)** Decision curve analysis for Models 1–3 across prespecified 5-year risk thresholds from 1% to 10%. The horizontal axis represents threshold probability and the vertical axis represents net benefit; treat-all and treat-none strategies are shown as reference curves. Model 1 included the 11 prespecified clinical predictors, Model 2 added binary carotid plaque presence, and Model 3 replaced binary plaque presence with the 0–6 simplified carotid plaque score. AUC, area under the receiver operating characteristic curve; CI, confidence interval; DCA, decision curve analysis; MACE, major adverse cardiovascular and cerebrovascular events.

### 3.7 Sensitivity and exploratory subgroup analyses

Prespecified sensitivity analyses yielded results consistent with the primary analysis (Table 6). The plaque-score effect remained similar after exclusion of MACE occurring within 90 days (HR, 1.30; 95% CI, 1.19–1.42), restriction to patients with complete archived PACS images (HR, 1.34; 95% CI, 1.22–1.47), exclusion of carotid stenosis ≥50% (HR, 1.29; 95% CI, 1.17–1.42), and exclusion of baseline AF (HR, 1.31; 95% CI, 1.20–1.44), with corresponding C-indices ranging from 0.793 to 0.806. Similar estimates were obtained in the Fine–Gray analysis (sHR, 1.31; 95% CI, 1.20–1.43), complete-case analysis (HR, 1.33; 95% CI, 1.21–1.46), and analysis using expanded MACE (HR, 1.28; 95% CI, 1.19–1.37). When the myocardial infarction component of hard MACE was restricted to type 1 myocardial infarction, 103 primary events remained and the plaque-score association was essentially unchanged (HR, 1.33; 95% CI, 1.21–1.46), with a C-index of 0.805 and a 5-year AUC of 0.808. The high-continuity follow-up analysis included 1,986 patients and 94 primary events and similarly yielded an HR of 1.31 (95% CI, 1.19–1.44), a C-index of 0.803, and a 5-year AUC of 0.806.

**Table 6:** Sensitivity analyses of the final prediction model.

Analysis	N	Events, n	Plaque score effect, HR or sHR (95% CI)	C-index (95% CI)	5-year AUC (95% CI)	5-year Brier score
Primary analysis	2,413	108	1.32 (1.21–1.44)	0.804 (0.765–0.843)	0.807 (0.768–0.846)	0.0381
Excluding MACE within 90 days	2,405	100	1.30 (1.19–1.42)	0.799 (0.758–0.840)	0.801 (0.760–0.842)	0.0358
Complete archived PACS images	1,876	88	1.34 (1.22–1.47)	0.806 (0.763–0.849)	0.809 (0.766–0.852)	0.0390
Excluding carotid stenosis ≥50%	2,291	92	1.29 (1.17–1.42)	0.793 (0.750–0.836)	0.795 (0.752–0.838)	0.0356
Excluding baseline AF	2,357	99	1.31 (1.20–1.44)	0.801 (0.760–0.842)	0.804 (0.763–0.845)	0.0367
Fine–Gray competing-risk analysis	2,413	108^1^	1.31 (1.20–1.43)^1^	—	0.803 (0.763–0.843)	0.0384
Complete-case analysis	2,176	96	1.33 (1.21–1.46)	0.802 (0.760–0.844)	0.805 (0.763–0.847)	0.0387
Expanded MACE outcome	2,413	153	1.28 (1.19–1.37)	0.784 (0.750–0.818)	0.790 (0.756–0.824)	0.0524
MI component restricted to type 1 MI	2,413	103	1.33 (1.21–1.46)	0.805 (0.765–0.845)	0.808 (0.769–0.847)	0.0365
High-continuity follow-up	1,986	94	1.31 (1.19–1.44)	0.803 (0.759–0.847)	0.806 (0.762–0.850)	0.0391

The same prespecified Model 3 predictor structure and coding were used in all sensitivity analyses without data-driven predictor selection. Model coefficients were re-estimated within the corresponding sensitivity-analysis dataset or outcome framework. Performance estimates shown for the primary analysis are apparent estimates to permit direct comparison with the sensitivity analyses; optimism-corrected performance is reported in Supplementary Table S5. ^1^For the Fine–Gray analysis, 108 primary MACE events were treated as events of interest and 62 non-cardiovascular deaths as competing events; the plaque-score effect is therefore reported as an sHR. The 5-year AUC and Brier score for this analysis were estimated within the competing-risk framework, and a conventional Cox C-index is not reported. In the type 1 myocardial infarction sensitivity analysis, type 2 and procedure-related myocardial infarctions were not counted as primary events; patients remained at risk for a subsequent qualifying cardiovascular death or ischemic stroke within the original 5-year follow-up period. High-continuity follow-up was defined as no interval longer than 18 months between successive accessible clinical encounters and, among patients remaining alive and free of MACE, a final accessible clinical record within 12 months of the 5-year follow-up horizon. AUC, area under the receiver operating characteristic curve; AF, atrial fibrillation; CI, confidence interval; HR, hazard ratio; MACE, major adverse cardiovascular and cerebrovascular events; MI, myocardial infarction; PACS, picture archiving and communication system; sHR, subdistribution hazard ratio.

In the calendar-time analysis, 40 primary MACE events occurred during pre-pandemic person-time and 68 occurred on or after March 11, 2020. The association per 1-point increase in plaque score was similar before (HR, 1.34; 95% CI, 1.19–1.50) and during the COVID-19 era (HR, 1.31; 95% CI, 1.18–1.44), with no clear evidence of plaque score   ×   calendar-period interaction (*P* = 0.721). When the myocardial infarction component was restricted to type 1 myocardial infarction, the corresponding HRs were 1.35 (95% CI, 1.20–1.52) and 1.31 (95% CI, 1.18–1.46), respectively, with no clear evidence of interaction (*P* = 0.742) (Supplementary Table S6). Four of the five adjudicated type 2 myocardial infarctions occurred during COVID-19-era follow-up.

When the established Model 3 was applied without coefficient refitting or recalibration to calendar-defined 3-year evaluation cohorts, the 3-year AUC was 0.808 (95% CI, 0.730–0.886) for prediction windows occurring entirely before the pandemic transition and 0.793 (95% CI, 0.682–0.904) for prediction windows beginning during the COVID-19 era. The corresponding calibration slopes were 0.97 and 0.91, and calibration-in-the-large values were −0.03 and 0.10, respectively. The COVID-19-era performance estimates were imprecise because only 17 primary events occurred in that cohort (Supplementary Table S7).

When the established Model 3 was applied without coefficient refitting, the C-index was 0.796 (95% CI, 0.753–0.839) in men and 0.807 (95% CI, 0.744–0.870) in women, and 0.781 (95% CI, 0.698–0.864) in patients with diabetes and 0.800 (95% CI, 0.758–0.842) in those without diabetes. There was no clear statistical evidence of interaction between plaque score and sex (*P* = 0.702) or diabetes status (*P* = 0.684); these analyses were exploratory and hypothesis-generating (Supplementary Table S8).

## 4 Discussion

In this retrospective hospital-based primary-prevention cohort, a simplified six-segment carotid plaque score was independently associated with the 5-year risk of hard major adverse cardiovascular and cerebrovascular events and provided incremental predictive information beyond conventional clinical factors and binary plaque presence. The final model retained good discrimination after bootstrap correction, showed generally acceptable calibration, and demonstrated potential clinical utility across relevant risk thresholds, with broadly similar findings across sensitivity analyses. The present study extends prior evidence by evaluating a pragmatic multivariable model incorporating this simplified plaque-extent measure in a hospital-based Chinese primary-prevention population, with explicit assessment of incremental performance, calibration, prediction error, and decision-analytic utility.

Carotid plaque was detected in 64.6% of participants, whereas only 5.1% had carotid stenosis ≥50%, indicating that ultrasonography predominantly identified subclinical rather than obstructive atherosclerosis. The plaque prevalence was substantially higher than the 21.0% reported in a nationwide Chinese health check-up population (3). However, the present cohort was older and was derived from a specialized hospital setting in which only 24.5% of examinations were performed for routine health evaluation, whereas 44.7% were performed for cardiovascular risk assessment and 30.9% during stable chronic disease management. Consistent with this referral enrichment, plaque prevalence increased from 41.4% in the routine health evaluation group to 66.1% in the cardiovascular risk assessment group and 80.9% in the stable chronic disease management group. The higher overall plaque burden should therefore be interpreted as a feature of the source population rather than as an estimate of population prevalence. Current European prevention guidance recognizes carotid plaque as a potential cardiovascular risk modifier, particularly when conventional risk estimates remain uncertain (8), and the American Society of Echocardiography has emphasized standardized carotid plaque assessment for cardiovascular risk characterization (20). Plaque occurred most frequently at the carotid bulbs, consistent with observations in asymptomatic Korean adults (21). Differences in age, referral setting, cardiovascular risk burden, and ultrasound protocols are likely to contribute to variation across cohorts.

A graded risk pattern was observed across plaque-score categories, with 5-year hard MACE incidences of 1.5%, 6.1%, and 15.4% for scores of 0–1, 2–4, and 5–6, respectively. This pattern is consistent with previous evidence that greater carotid plaque extent carries prognostic information beyond binary plaque presence. However, plaque extent should not be interpreted as equivalent to carotid stenosis severity. Although the prevalence of stenosis ≥50% increased with higher plaque scores, 87.1% of patients with scores of 5–6 still had maximal stenosis <50%. Conversely, some patients with low plaque scores had clinically relevant focal stenosis. The simplified score therefore primarily captures the distribution of atherosclerotic plaque across carotid segments rather than the hemodynamic severity of a single lesion. Sillesen et al. similarly reported that greater carotid plaque burden and maximal plaque thickness were associated with progressively higher cardiovascular event risk in asymptomatic adults (22). In the population-based ACE 1950 study, carotid plaque score predicted ischemic stroke and MACE after adjustment for SCORE2 (18). Matangi et al. used a comparable 0–6 score across the bilateral common carotid arteries, bulbs, and internal carotid arteries and reported 10-year MACE rates of 4.1%, 8.7%, and 20.0% for scores <2, 2–4, and 5–6, respectively (11). Although absolute risks cannot be directly compared because of differences in follow-up and population characteristics, the graded association is consistent. Ischemic stroke accounted for half of the first hard MACE events in our cohort. This should not imply that individual carotid plaques directly caused these events, because carotid plaque also reflects systemic atherosclerotic burden and may identify risk across multiple vascular territories.

Older age, higher systolic blood pressure, current smoking, and lower eGFR remained independently associated with MACE in the final model, consistent with established cardiovascular risk relationships (8). Each 1-point increase in plaque score was associated with a 32% higher relative hazard of hard MACE after adjustment for the prespecified clinical predictors. Bao et al. demonstrated that adding carotid plaque to SCORE2 improved cardiovascular event prediction in individuals without established cardiovascular disease (23). A recent systematic review likewise concluded that carotid ultrasound may improve primary-prevention risk stratification, although substantial heterogeneity exists in plaque definitions and assessment methods (24). The plaque score may therefore capture cumulative exposure to measured and unmeasured atherogenic processes that is not fully represented by individual baseline risk factors. Conversely, the lack of statistical significance for BMI, LDL-C, HDL-C, baseline statin use, and antihypertensive treatment should not be interpreted as evidence that these factors are clinically unimportant. Baseline measurements do not capture treatment duration, adherence, subsequent intensification, or longitudinal risk-factor control.

The model-level comparisons provide the more specific contribution of the present study. Adding any plaque to the clinical model increased the apparent C-index from 0.753 to 0.776, whereas incorporation of the 0–6 score increased it to 0.804, with corresponding improvements in time-dependent AUCs and Brier score. Similar incremental value has been reported with other measures of carotid plaque burden. Adams et al. showed improved risk prediction when ultrasound-derived plaque burden was added to the PROCAM equation (25), while an ARCO analysis found that total plaque area added prognostic information to SCORE2 (26). Sánchez-Bacaicoa et al. reported that a plaque-burden scale based on the number of affected carotid segments was independently associated with first MACE and improved discrimination when combined with SCORE2 (27). However, different plaque metrics are not interchangeable. Genkel et al. found that total plaque area, rather than plaque score or maximal thickness, independently predicted subsequent cardiovascular and cerebrovascular events (28). Continuous measures may preserve more information, whereas a segment-based score sacrifices some anatomical detail in favor of simplicity and feasibility. Likewise, duplex-defined stenosis severity represents a distinct vascular phenotype that incorporates luminal narrowing and flow hemodynamics rather than simply the number of plaque-positive segments. The present score can be derived from six routinely examined carotid segments without additional plaque tracing, volumetric analysis, or incorporation of Doppler velocity measurements into the score itself. Consistent with this pragmatic approach, the ICEBERG-3 study also demonstrated prognostic value from a six-segment carotid plaque score when combined with stress echocardiographic assessment (29). These incremental improvements should nevertheless be interpreted within the context of a single development cohort. They do not establish that the six-segment score is superior to other quantitative plaque measures or to contemporary guideline-based risk algorithms, which were not directly compared in the present study. The score is therefore best regarded as a pragmatic candidate imaging marker whose incremental value requires confirmation in external populations.

The high interobserver agreement further supports the feasibility of this simplified approach. Segment-level *κ* values ranged from 0.84 to 0.89, and the ICC for the overall plaque score was 0.94. Standardized plaque assessment is important because ultrasound-based measurements can vary with scanning planes, plaque definitions, acquisition techniques, and reader experience (20). Importantly, restriction to patients with complete archived PACS images produced an almost identical plaque-score association and similar model discrimination. Nevertheless, reproducibility within a single center does not ensure equivalent performance across laboratories. External studies using different equipment, operators, and reporting protocols are needed to confirm measurement robustness.

Internal validation suggested only modest optimism. The C-index decreased from 0.804 to 0.792 after bootstrap correction, while the optimism-corrected calibration slope was 0.93. Modest overprediction was observed at the upper end of predicted risk. Calibration is particularly important when absolute risk estimates are intended to inform clinical decisions (30). Decision curve analysis showed greater net benefit for the final model than for the clinical and binary-plaque models across most thresholds between 2% and 10%. Because DCA incorporates the relative consequences of false-positive and false-negative classifications, it provides information beyond discrimination alone (31). These findings suggest potential value for identifying individuals who may warrant more intensive preventive assessment among comparable hospital-based primary-prevention patients in whom carotid ultrasonography has already been obtained. The model was not developed as a population-screening instrument or as a tool for selecting individuals for carotid ultrasonography. Because model discrimination and calibration depend partly on the case mix and baseline risk of the development population, performance in lower-risk community or primary-care populations cannot be inferred from the present internal validation. However, DCA does not demonstrate that using the model to guide treatment improves outcomes, and prospective impact studies remain necessary.

The extensive sensitivity analyses support the robustness of the principal findings. Excluding MACE occurring within 90 days yielded a similar plaque-score association, reducing concern that the primary result was driven by imminent but clinically unrecognized disease. Excluding carotid stenosis ≥50% also produced comparable estimates, indicating that predictive information was not restricted to advanced obstructive disease. Results remained similar after excluding baseline atrial fibrillation, applying a Fine–Gray competing-risk model, restricting analysis to complete cases, and using expanded MACE as the outcome. The association and model discrimination were also materially unchanged when the myocardial infarction component was restricted to type 1 myocardial infarction and when the analysis was restricted to patients with high-continuity longitudinal follow-up.

The COVID-19 pandemic warrants separate consideration because healthcare utilization and the mechanisms underlying cardiovascular presentations changed during this period, while severe infection, hypoxemia, inflammation, and hemodynamic instability may contribute to myocardial injury and type 2 myocardial infarction (32, 33). In the calendar-time analysis, the association between plaque score and MACE was similar before and during the COVID-19 era, with no clear evidence of interaction; the same pattern was observed when the myocardial infarction component was restricted to type 1 myocardial infarction. When the original Model 3 was applied without refitting, discrimination was broadly similar across calendar-defined 3-year prediction cohorts, whereas the COVID-19-era cohort showed a modestly lower calibration slope and slight underprediction on average. However, only 17 primary events contributed to the COVID-19-era performance analysis, resulting in wide confidence intervals. These analyses therefore provide no clear evidence of a major change in the plaque-score association across the pandemic transition but do not establish equivalent predictive performance or calibration across calendar periods.

The frozen model yielded broadly similar discrimination point estimates in men and women and in patients with and without diabetes. Previous studies have likewise demonstrated prognostic information from carotid plaque across heterogeneous primary-prevention populations (11, 18, 23). However, these subgroup analyses were exploratory and hypothesis-generating because only 38 primary events occurred among women and 28 among patients with diabetes. The resulting confidence intervals were relatively wide, and the interaction tests had limited statistical power. Accordingly, the absence of statistically significant interaction should not be interpreted as evidence of equivalent model performance across sex or diabetes subgroups. Larger external cohorts should assess whether discrimination, calibration, or clinically useful thresholds differ according to sex, diabetes, age, renal function, or background preventive therapy.

Several limitations should be acknowledged. First, this was a retrospective single-center study conducted in a specialized cardiovascular and cerebrovascular hospital, with enrichment for patients undergoing cardiovascular risk assessment or stable chronic disease management. The 64.6% prevalence of carotid plaque therefore should not be considered representative of the general Chinese primary-prevention population, and the case mix may have influenced absolute risk and model performance. Residual selection effects also remain possible despite comparison with patients excluded for incomplete plaque assessment or unavailable follow-up. Second, although the prespecified sample-size target was met, only 108 primary events occurred, limiting precision for individual coefficients, interaction tests, and more complex predictor functions. Third, risk factors and medications were assessed primarily at baseline, and subsequent changes in treatment, smoking, glycemic control, or other preventive interventions were not incorporated. Fourth, the simplified score measures plaque extent rather than comprehensive carotid disease severity and does not capture luminal stenosis, plaque area or volume, composition, echogenicity, calcification, ulceration, or progression. Although stenosis grading followed established duplex criteria, whole-cohort categories were derived from historical reports and exact PSV, EDV, and ICA/CCA PSV ratios were not consistently available for retrospective analysis. More detailed standardized vascular phenotyping may provide additional prognostic information (20, 28). Fifth, ascertainment of non-fatal events depended on information accessible to the study hospital. Although cardiovascular deaths were supplemented by the regional mortality registry, non-fatal myocardial infarction or ischemic stroke occurring entirely outside the accessible healthcare system could have been missed. The high ascertainment completeness and similar high-continuity follow-up results reduce, but do not eliminate, this concern. Follow-up also overlapped the COVID-19 pandemic; calendar-time and frozen-model analyses showed no marked change in the plaque-score association or model performance, but the limited number of pandemic-era events precludes firm conclusions regarding temporal equivalence. Sixth, non-cardiovascular death was censored in the primary Cox model, although Fine–Gray analyses produced similar results. Finally, the model underwent internal validation only. Bootstrap correction addresses optimism within the development cohort but does not establish transportability. External validation in populations with different referral patterns and baseline risk, together with direct comparison against contemporary risk algorithms and prospective evaluation of clinical impact, is required before routine implementation.

## 5 Conclusion

In this hospital-based primary-prevention cohort of adults aged ≥40 years already undergoing carotid ultrasonography, a simplified six-segment carotid plaque score provided incremental predictive information for 5-year hard MACE beyond conventional clinical factors and binary plaque presence. The score showed high interobserver reproducibility and retained most of its discrimination after bootstrap correction. These findings extend previous evidence on carotid plaque extent by providing internally validated model-level estimates of discrimination, calibration, prediction error, and clinical net benefit in this clinical setting. They do not establish superiority over other plaque-burden measures or established cardiovascular risk algorithms. External validation in populations with different referral patterns and baseline risk is required before broader clinical application.

## Data Availability

The raw data supporting the conclusions of this article will be made available by the authors, without undue reservation.
