# Supplementary material for: Development and internal validation of a 5-year major cardiovascular and cerebrovascular events prediction model using a simplified carotid plaque score in a hospital-based primary-prevention cohort
Source: Front Cardiovasc Med. 2026 Oct 28;13:1966647. doi: 10.3389/fcvm.2026.1966647 (PMC13751385; doi:10.3389/fcvm.2026.1966647)
Supplement: Supplementary file 1 [file Datasheet1.docx]

**Supplementary Table S1.** Examination setting, referral source, and cardiovascular risk characteristics according to the primary indication for carotid ultrasonography

| **Characteristic** | **Overall (N = 2,413)** | **Routine health evaluation (n = 590)** | **Cardiovascular risk assessment (n = 1,078)** | **Stable chronic disease management (n = 745)** |
| --- | --- | --- | --- | --- |
| **Encounter setting** |  |  |  |  |
| Outpatient | 1,681 (69.7) | 590 (100.0) | 849 (78.8) | 242 (32.5) |
| Inpatient | 732 (30.3) | 0 (0.0) | 229 (21.2) | 503 (67.5) |
| **Referral source** |  |  |  |  |
| Health management/physical examination center | 590 (24.5) | 550 (93.2) | 40 (3.7) | 0 (0.0) |
| Cardiovascular medicine | 883 (36.6) | 20 (3.4) | 500 (46.4) | 363 (48.7) |
| Cerebrovascular/neurology service | 540 (22.4) | 5 (0.8) | 330 (30.6) | 205 (27.5) |
| Other internal medicine service | 400 (16.6) | 15 (2.5) | 208 (19.3) | 177 (23.8) |
| **Clinical characteristics** |  |  |  |  |
| Age, years | 59.8 ± 9.7 | 53.4 ± 8.1 | 59.6 ± 8.7 | 65.2 ± 9.0 |
| Male sex | 1,237 (51.3) | 295 (50.0) | 557 (51.7) | 385 (51.7) |
| BMI, kg/m² | 24.8 ± 3.2 | 24.0 ± 2.9 | 24.8 ± 3.1 | 25.5 ± 3.4 |
| Systolic blood pressure, mmHg | 130.9 ± 15.8 | 124.8 ± 13.8 | 130.9 ± 15.0 | 135.8 ± 16.8 |
| Current smoking | 379 (15.7) | 70 (11.9) | 174 (16.1) | 135 (18.1) |
| Diabetes mellitus | 362 (15.0) | 29 (4.9) | 129 (12.0) | 204 (27.4) |
| Antihypertensive medication use | 769 (31.9) | 76 (12.9) | 278 (25.8) | 415 (55.7) |
| Baseline statin use | 641 (26.6) | 59 (10.0) | 249 (23.1) | 333 (44.7) |
| LDL-C, mmol/L | 2.93 ± 0.76 | 2.89 ± 0.72 | 2.96 ± 0.77 | 2.93 ± 0.78 |
| HDL-C, mmol/L | 1.28 ± 0.31 | 1.31 ± 0.29 | 1.28 ± 0.31 | 1.26 ± 0.33 |
| eGFR, mL/min/1.73 m² | 87.5 ± 17.5 | 92.6 ± 15.2 | 88.3 ± 16.6 | 82.2 ± 18.4 |
| AF | 56 (2.3) | 4 (0.7) | 16 (1.5) | 36 (4.8) |
| **Carotid ultrasound characteristics** |  |  |  |  |
| Any carotid plaque | 1,560 (64.6) | 244 (41.4) | 713 (66.1) | 603 (80.9) |
| Bilateral carotid plaque | 650 (26.9) | 64 (10.8) | 287 (26.6) | 299 (40.1) |
| Simplified carotid plaque score | 1 (0–3) | 0 (0–1) | 1 (0–3) | 2 (1–4) |
| Maximal carotid stenosis ≥50% | 122 (5.1) | 8 (1.4) | 45 (4.2) | 69 (9.3) |

Notes: Values are presented as mean ± SD, median (IQR), or n (%), as appropriate. Percentages within the indication-specific columns use the corresponding indication group as the denominator. The primary indication for carotid ultrasonography was determined from the ultrasound order and index-encounter documentation. Cardiovascular risk assessment referred to examination performed for evaluation of cardiovascular or cerebrovascular risk in patients without established clinical cardiovascular or cerebrovascular disease; stable chronic disease management referred to examination obtained during ongoing management of chronic cardiovascular risk conditions without an acute cardiovascular or cerebrovascular presentation. Referral source represents the service associated with the index ultrasound order. Values for variables with missing data were calculated from available observations. Comparisons were descriptive; no hypothesis testing was performed across indication groups. AF, atrial fibrillation; BMI, body mass index; eGFR, estimated glomerular filtration rate; HDL-C, high-density lipoprotein cholesterol; LDL-C, low-density lipoprotein cholesterol; SD, standard deviation.

**Supplementary Table S2.** Comparison of available baseline characteristics between the analyzed cohort and patients excluded because of incomplete plaque assessment or unavailable outcome ascertainment

| **Variable** | **Analyzed cohort (n = 2,413)** | **Incomplete plaque assessment (n = 128)** | **Unavailable outcome ascertainment (n = 71)** | **Statistic** | **P value** |
| --- | --- | --- | --- | --- | --- |
| Age, years | 59.8 ± 9.7 | 62.2 ± 10.4 | 58.1 ± 11.2 | F = 4.78 | 0.008 |
| Male sex | 1,237 (51.3) | 67 (52.3) | 34 (47.9) | χ² = 0.38 | 0.826 |
| BMI, kg/m² | 24.8 ± 3.2 | 24.9 ± 3.3 | 24.6 ± 3.4 | F = 0.20 | 0.823 |
| Systolic blood pressure, mmHg | 130.9 ± 15.8 | 132.8 ± 16.6 | 129.9 ± 17.4 | F = 1.04 | 0.354 |
| Current smoking | 379 (15.7) | 18 (14.1) | 13 (18.3) | χ² = 0.62 | 0.732 |
| Diabetes mellitus | 362 (15.0) | 25 (19.5) | 8 (11.3) | χ² = 2.79 | 0.248 |
| Antihypertensive medication use | 769 (31.9) | 46 (35.9) | 18 (25.4) | χ² = 2.36 | 0.307 |
| Baseline statin use | 641 (26.6) | 29 (22.7) | 14 (19.7) | χ² = 2.54 | 0.281 |
| AF | 56 (2.3) | 5 (3.9) | 1 (1.4) | χ² = 1.61 | 0.447 |

Notes: Values are presented as mean ± SD or n (%). Continuous variables were compared across the three groups using one-way analysis of variance, and categorical variables were compared using χ² tests. Comparisons were descriptive and were performed using available baseline data.

**Supplementary Table S3.** Relationship between the simplified carotid plaque score and maximal carotid stenosis severity

| **Simplified carotid plaque score** | **N** | **<50%, n (%)** | **50%–69%, n (%)** | **70%–99%, n (%)** | **Occlusion, n (%)** | **Stenosis ≥50%, n (%)** |
| --- | --- | --- | --- | --- | --- | --- |
| 0 | 853 | 853 (100.0) | 0 (0.0) | 0 (0.0) | 0 (0.0) | 0 (0.0) |
| 1 | 411 | 388 (94.4) | 18 (4.4) | 5 (1.2) | 0 (0.0) | 23 (5.6) |
| 2 | 337 | 318 (94.4) | 17 (5.0) | 2 (0.6) | 0 (0.0) | 19 (5.6) |
| 3 | 318 | 294 (92.5) | 19 (6.0) | 4 (1.3) | 1 (0.3) | 24 (7.5) |
| 4 | 245 | 221 (90.2) | 20 (8.2) | 4 (1.6) | 0 (0.0) | 24 (9.8) |
| 5 | 154 | 136 (88.3) | 14 (9.1) | 3 (1.9) | 1 (0.6) | 18 (11.7) |
| 6 | 95 | 81 (85.3) | 10 (10.5) | 3 (3.2) | 1 (1.1) | 14 (14.7) |
| **Overall** | **2,413** | **2,291 (94.9)** | **98 (4.1)** | **21 (0.9)** | **3 (0.1)** | **122 (5.1)** |

Notes: Values are n (%) within each plaque-score category. The simplified carotid plaque score represents the number of plaque-positive segments across the bilateral CCAs, carotid bulbs, and proximal ICAs and therefore reflects plaque extent rather than luminal stenosis severity. Maximal carotid stenosis refers to the greatest stenosis grade documented in either carotid artery at the index examination. Stenosis severity was classified using the institutional duplex protocol based on the Society of Radiologists in Ultrasound consensus criteria, incorporating gray-scale and color Doppler assessment with ICA PSV and supporting ICA EDV and ICA/CCA PSV ratio measurements. Normal arteries were included in the <50% category. Percentages may not total 100% because of rounding. CCA, common carotid artery; EDV, end-diastolic velocity; ICA, internal carotid artery; PSV, peak systolic velocity.

**Supplementary Table S4.** Interobserver reliability of carotid ultrasound assessments

| **Ultrasound assessment** | **Reassessment sample, n** | **Reliability statistic** | **95% CI** |
| --- | --- | --- | --- |
| Any carotid plaque | 483 | κ = 0.91 | 0.87–0.95 |
| Left CCA plaque | 483 | κ = 0.86 | 0.81–0.91 |
| Right CCA plaque | 483 | κ = 0.84 | 0.78–0.89 |
| Left carotid bulb plaque | 483 | κ = 0.89 | 0.85–0.93 |
| Right carotid bulb plaque | 483 | κ = 0.88 | 0.83–0.92 |
| Left ICA plaque | 483 | κ = 0.85 | 0.80–0.90 |
| Right ICA plaque | 483 | κ = 0.84 | 0.79–0.89 |
| Bilateral carotid plaque | 483 | κ = 0.90 | 0.85–0.94 |
| Maximal carotid stenosis ≥50% | 483 | κ = 0.88 | 0.80–0.95 |
| Simplified carotid plaque score | 483 | ICC = 0.94 | 0.93–0.95 |
| Total plaque number | 483 | ICC = 0.91 | 0.89–0.93 |
| Maximal plaque thickness, mm¹ | 313 | ICC = 0.92 | 0.90–0.94 |

Notes: The reassessment sample comprised 483 randomly selected patients, representing 20.0% of the analytic cohort. Cohen κ was used for categorical assessments, and ICC was used for quantitative ultrasound assessments. ¹Reliability of maximal plaque thickness was assessed among patients with measurable plaque in the reassessment sample. CI, confidence interval; ICC, intraclass correlation coefficient.

**Supplementary Table S5.** Apparent and optimism-corrected performance of the three prediction models

| **Performance measure** | **Model 1** | **Model 2** | **Model 3** |
| --- | --- | --- | --- |
| Harrell C-index |  |  |  |
| Apparent | 0.753 | 0.776 | 0.804 |
| Optimism-corrected | 0.744 | 0.765 | 0.792 |
| Estimated optimism | 0.009 | 0.011 | 0.012 |
| 5-year Brier score |  |  |  |
| Apparent | 0.0417 | 0.0402 | 0.0381 |
| Optimism-corrected | 0.0424 | 0.0411 | 0.0393 |
| Optimism-related increase | 0.0007 | 0.0009 | 0.0012 |
| Calibration slope |  |  |  |
| Apparent | 1.00 | 1.00 | 1.00 |
| Optimism-corrected | 0.95 | 0.94 | 0.93 |

Notes: Apparent performance was estimated in the full development cohort. Optimism correction was based on 1,000 patient-level bootstrap resamples, with the prespecified missing-data and modeling procedures incorporated into the validation workflow. For the C-index, estimated optimism represents the difference between apparent and optimism-corrected performance. For the Brier score, the optimism-related increase represents the increase in prediction error after optimism correction. Lower Brier scores indicate lower overall prediction error. The calibration slope reflects agreement between predicted and observed relative risks, with values below 1 indicating some degree of overfitting. Model 1 included the 11 prespecified clinical predictors; Model 2 added any carotid plaque to Model 1; and Model 3 added the 0–6 simplified carotid plaque score to Model 1.

**Supplementary Table S6.** Association of the simplified carotid plaque score with MACE before and during the COVID-19 era

| **Outcome definition** | **Pre-pandemic events, n** | **Plaque score HR before March 11, 2020 (95% CI)** | **COVID-19-era events, n** | **Plaque score HR on or after March 11, 2020 (95% CI)** | **P for interaction** |
| --- | --- | --- | --- | --- | --- |
| Primary hard MACE | 40 | 1.34 (1.19–1.50) | 68 | 1.31 (1.18–1.44) | 0.721 |
| Hard MACE with MI component restricted to type 1 MI | 39 | 1.35 (1.20–1.52) | 64 | 1.31 (1.18–1.46) | 0.742 |

Notes: Follow-up was split at March 11, 2020, when the World Health Organization characterized COVID-19 as a pandemic. Calendar period was treated as a time-varying indicator in an extended Cox model in counting-process form. The model retained the same covariate structure as Model 3 and included a simplified carotid plaque score × calendar-period interaction term. Hazard ratios represent the association with MACE per 1-point increase in the simplified carotid plaque score within the corresponding calendar period. In the type 1 MI analysis, type 2 and procedure-related myocardial infarctions did not constitute an event unless another qualifying hard-MACE component subsequently occurred. The interaction P value tests whether the plaque-score association differed between pre-pandemic and COVID-19-era person-time. CI, confidence interval; HR, hazard ratio; MACE, major adverse cardiovascular and cerebrovascular events; MI, myocardial infarction.

**Supplementary Table S7.** Performance of the frozen Model 3 in calendar-defined 3-year prediction cohorts before and during the COVID-19 era

| **Performance measure** | **Entirely pre-pandemic prediction window** | **COVID-19-era prediction window** |
| --- | --- | --- |
| Index examination period | Jan 1, 2015–Mar 10, 2017 | Mar 11–Dec 31, 2020 |
| N | 804 | 368 |
| Primary MACE within 3 years, n | 24 | 17 |
| Mean predicted 3-year risk, % | 3.1 | 4.3 |
| Observed 3-year risk, % | 3.2 | 4.7 |
| 3-year time-dependent AUC (95% CI) | 0.808 (0.730–0.886) | 0.793 (0.682–0.904) |
| 3-year Brier score | 0.0278 | 0.0411 |
| Calibration-in-the-large (95% CI) | −0.03 (−0.31 to 0.25) | 0.10 (−0.28 to 0.48) |
| Calibration slope (95% CI) | 0.97 (0.69–1.25) | 0.91 (0.56–1.26) |

Notes: Model 3 coefficients and the corresponding 3-year baseline survival estimate derived from the full development cohort were applied unchanged in both calendar-defined cohorts; no predictor reselection, coefficient refitting, or era-specific recalibration was performed. The pre-pandemic cohort included patients whose complete 3-year prediction horizon ended before March 11, 2020. The COVID-19-era cohort included patients whose index examination occurred on or after March 11, 2020. Patients whose 3-year prediction horizons crossed March 11, 2020 were excluded from this performance comparison only. Observed 3-year risks and performance measures accounted for censoring. Calibration-in-the-large values below or above 0 indicate overall overprediction or underprediction, respectively. AUC, area under the receiver operating characteristic curve; CI, confidence interval; MACE, major adverse cardiovascular and cerebrovascular events.

**Supplementary Table S8.** Prespecified exploratory subgroup performance of the final prediction model

| **Subgroup** | **N** | **Primary MACE, n** | **C-index (95% CI)** | **P for interaction** |
| --- | --- | --- | --- | --- |
| Male | 1,237 | 70 | 0.796 (0.753–0.839) | 0.702¹ |
| Female | 1,176 | 38 | 0.807 (0.744–0.870) | — |
| Diabetes mellitus | 362 | 28 | 0.781 (0.698–0.864) | 0.684² |
| No diabetes mellitus | 2,051 | 80 | 0.800 (0.758–0.842) | — |

Notes: The established Model 3 was applied directly to each subgroup using the coefficients and 5-year baseline survival estimate derived from the full cohort; no predictor reselection or coefficient refitting was performed within subgroups. C-indices describe discrimination of the frozen model within each subgroup. ¹P value for the simplified carotid plaque score × sex interaction. ²P value for the simplified carotid plaque score × diabetes-status interaction. Interaction tests were performed in the full cohort with the corresponding interaction term added to the prespecified Model 3 covariates. CI, confidence interval.
